# Effect of Cooling Method on Physical and Mechanical Properties of PVA Fiber-Reinforced High-Strength Concrete Exposed to High Temperature

**DOI:** 10.3390/polym16162286

**Published:** 2024-08-13

**Authors:** Jian Wu, Yuxi Wang, Chaoqun Hu, Liangjie Hu, Lidan Zhang, Jianhui Wang, Weigao Ding

**Affiliations:** 1 Shaanxi Key Laboratory of Safety and Durability of Concrete Structures, Xijing University, Xi’an 710123, China; wujian2085@126.com (J.W.);; 2 School of Infrastructure Engineering, Dalian University of Technology, Dalian 116024, China

**Keywords:** high temperature, cooling methods, PVA fiber-reinforced HSC, mechanical properties, microscopic analysis

## Abstract

High-strength concrete (HSC) boasts excellent compressive strength and durability, making it a popular choice in various engineering applications. However, under the impact of high temperatures, HSC tends to crack easily, so it is combined with polyvinyl alcohol fiber (PVA fiber) to explore its engineering application prospect. This paper investigated the physical and mechanical characteristics of HSC reinforced with PVA fibers subjected to different heating temperatures and cooling techniques. The experimental results reveal a correlation between rising temperatures and observable changes in the specimens: a progressively lighter surface hue, an augmented frequency of cracking, and a considerable escalation in the mass loss rate, particularly after the temperature exceeds 400 °C. Regarding mechanical properties, the dynamic elastic modulus and compressive and flexural strength all decrease as the heating temperature increases. As the amount of PVA fiber rises while maintaining a steady temperature, these measurements initially show an increase followed by a decrease. The fiber contents yielding the best compressive and flexural strength are 0.2% and 0.3%, in that order. Considering the influence of cooling methods, water spray cooling has a greater impact on physical and mechanical properties than natural cooling. Furthermore, SEM was employed to scrutinize the microstructure of HSC, enhancing comprehension of the alterations in its physical and mechanical characteristics. The findings of this research offer significant information regarding the high-temperature behavior of HSC, serving as a valuable resource for guiding the design, building, and upkeep of structures that incorporate HSC. Additionally, this study will aid in advancing the progress and utilization of HSC technology.

## 1. Introduction

HSC is a concrete material with high strength and durability, and widely used in buildings, bridges, tunnels, and other engineering projects, which can provide better structural performance and durability [1,2]. In practical engineering applications, reducing structure weight and saving raw materials can meet the strategic requirements of energy conservation and sustainable development [3]. However, the deformation ability of HSC is weak, and it is prone to cracking under high temperatures. Consequently, this article seeks to improve the physical and mechanical performance of HSC by integrating PVA fibers. Moreover, the cooling sequence of a structure constructed with HSC differs during a fire event. Hence, understanding how different cooling techniques affect HSC’s behavior at elevated temperatures is essential.

In recent years, research on HSC has mainly focused on the optimization of mix ratio and the application of additives, etc. By adjusting the water-to-binder ratio, utilizing various cement materials, incorporating mineral admixtures, and optimizing the mix ratio of the concrete, the performance of HSC can be effectively enhanced. Samir et al. [4] demonstrated that silica fume has a beneficial impact on concrete properties. Increasing the substitution level of silica fume significantly augments the mechanical characteristics of concrete and elevates its compressive strength. In addition, the research results of Rid [5] showed that incorporating fly ash can effectively reduce the cost of concrete and produce HSC by substituting a portion of the cement. While improving concrete raw materials, it is also necessary to add admixture. Malathy [6] found that replacing regular water with magnetized water in concrete could increase its compressive and split tensile strengths by 12% and 8.9%, enhancing its mechanical performance. When designing concrete mixtures, it is crucial to consider the water-to-cement ratio, cement quantity, and the choice of suitable water reducers [7]. Ghamari et al. [8] found that the weight loss decreased and compressive strength increased as the waste glass content increased. Consequently, previous studies have extensively explored the impact factors such as aggregates, minerals, cement content, and water/cement ratio on the properties of HSC. However, conventional mix proportions often result in elevated costs. To address this issue, this study incorporates water-reducing agents into the manufacturing process of HSC.

HSC has low shrinkage properties, and when subjected to external forces, it is easy to cause deformation in the component, causing it to rupture, while the addition of fibers can effectively address these shortcomings. Research by Shen et al. [9] investigated that increasing the PVA fiber content in HSC could delay cracking and enhance its performance. Another study by Shen et al. [10] demonstrated that incorporating PVA fibers into concrete improves tensile strength and decreases the tensile stress rate, thus reducing the likelihood of cracks in HSC. Moreover, PVA fibers reduce the concrete’s compressive modulus of elasticity and its propensity for dry shrinkage properties, with longer PVA fibers showing greater effectiveness [11]. Adding PVA fiber to concrete improved its compressive and flexural strength and also reduced the rate of mass loss as well as the relative dynamic elastic modulus after exposure to freeze–thaw cycles [12]. The study of Yao et al. [13] indicated that fiber-reinforced recycled aggregate had better performance than ordinary concrete under high temperature. Voutetaki et al. [14] used a structural health monitoring (SHM) system to detect damage in fiber-reinforced concrete. The results indicated that the fibers could improve the post-peak compressive behavior of concrete. Mpalaskas et al. [15] used the ultrasonic method and acoustic emission method to analyze the influence of the temperature on the cracking phenomenon of materials. They found that acoustic emission was quite sensitive to fracture incidents. These research findings illustrate that incorporating fibers can mitigate cracking in regular concrete and improve both the mechanical properties and microstructure of HSC. Despite this, the utilization of fibers in HSC remains somewhat constrained. Therefore, this study focuses on investigating PBA fiber-reinforced high-strength concrete.

Subjecting HSC to high temperature conditions leads to the evaporation of water within the material, consequently generating a significant temperature gradient. This gradient induces cracking, distortion, and diminished performance, ultimately jeopardizing the structure’s safety and dependability. As such, investigating the alterations in HSC’s physical and mechanical properties following high-temperature exposure and uncovering efficient cooling techniques holds immense engineering importance. Zhai et al. [16] examined how high-temperature cooling impacts concrete’s physical and mechanical properties in their research. The results showed that water cooling led to stronger specimens than natural cooling below 400 °C. In terms of compressive strength, the findings of Yuan et al. [17] showed that under natural cooling conditions, the residual compressive strength value declined as the heating temperature increased. Meanwhile, the results of Jing et al. [18] and Li [19] revealed that water-cooled specimens had lower compressive strength than naturally cooled ones, especially at higher temperatures. From a microscopic perspective, Zhao [20] utilized SEM observations to discover that the degree of bonding between the concrete aggregate and cement within the sample varied after being subjected to high temperature and cooling methods. These studies examined how the performance and microstructure of ordinary concrete change under various cooling systems following exposure to high temperatures. However, HSC, being denser, is more susceptible to cracking under high temperature conditions. Consequently, the effect of the cooling system becomes crucially important. Therefore, it is essential to evaluate how the temperature and the cooling system impact the performance of HSC.

Therefore, this paper primarily investigates the appearance, mass loss rate, dynamic elastic modulus, compressive strength, and flexural strength of specimens. It proposes regression analysis-based formulas for estimating the strength and temperature variations under different cooling methods. Additionally, this study analyzes the influence of varying heating temperatures on the composition of PVA fiber-reinforced HSC using SEM tests. It establishes connections between compositional changes and the macroscopic properties of PVA fiber-reinforced HSC. These findings aim to elucidate potential behaviors of fiber-reinforced HSC structures under different fire conditions, contributing to structural safety and design considerations.

## 2. Test Profile

### 2.1. Specimen Design and Preparation

In this paper, ordinary Portland cement with strength grade of P·O 52.5, which was produced by Shandong Province Zhucheng Jiuqi Building Materials limited company, was used to manufacture concrete. The coarse aggregate consisted of graded gravel ranging in particle size from 5 to 20 mm; the fine aggregate consisted of ordinary river sand, which has a fineness modulus of 2.63 and a packing density of 1480 kg/m^3^. During the mixing procedure, purified municipal tap water was utilized along with a water-reducing agent that achieved a 30% rate of water reduction, and Grade I fly ash with an apparent density of 2600 kg/m^3^, S95 grade slag powder, and silica fume with a water content of 1.8% and SiO_2_ content of 92.2% were added into the mixture. The PVA fiber was manufactured by Shanghai Kaiyuan Chemical Technology Co., Ltd. (Shanghai, China), and the melting point, length, diameter, and tensile strength of fibers provided by this compony were 200 °C, 12 mm, 15.09 μm, and 1830 MPa, respectively.

Based on the requirements of Chinese standard JGJ/T 281-2012 [21], the HSC strength grade was defined as C80. To enhance the performance of HSC, mineral admixtures were added and the water/binder ratio was adjusted to 0.2 by trial and error. Then, according to Chinese standard JGJ/T 221-2010 [22], corresponding adjustments were made in accordance with the fiber content in concrete, the fiber-reinforced concrete’s intended usage, and the mixture’s workability. Table 1 displays the specimen mix percentages. Based on the trials of HSC specimens, it can be observed that as the fiber content increases, the compressive strength of HSC shows an initial rise followed by a decline. Hence, in this study, the increment in fiber content during experimentation was set at 0.1%, and the maximum fiber content was 0.4%.

The production process of fiber-reinforced HSC specimens should refer to the methods specified in Chinese standards GB/T 50080-2016 [23] and CECS 13:2009 [24]. The concrete mixture was placed into cube molds measuring 100 mm × 100 mm × 100 mm to manufacture specimens for compression testing, whereas prismatic molds with a size of 100 mm × 100 mm × 400 mm were utilized to produce specimens for flexural testing. After the removal of mold, the specimens were subjected to 28 days curing process within the curing box. According to Chinese standard GB/T 50081-2002 [25], the dimensions of standard specimens used for cube compressive testing are 150 mm × 150 mm × 150 mm, while the flexural standard specimens have dimensions of 150 mm × 150 mm × 550 mm, so the measured strength value needed to be reduced, and the groups tested are depicted in Table 2.

### 2.2. High Temperature Cooling Test

The heating curves used in this study refer to the heating schedule utilized in Chinese standard GB/T 9978.1-2008 [26], which is applicable to concrete using fibers as reinforced materials.

**Figure 1:**
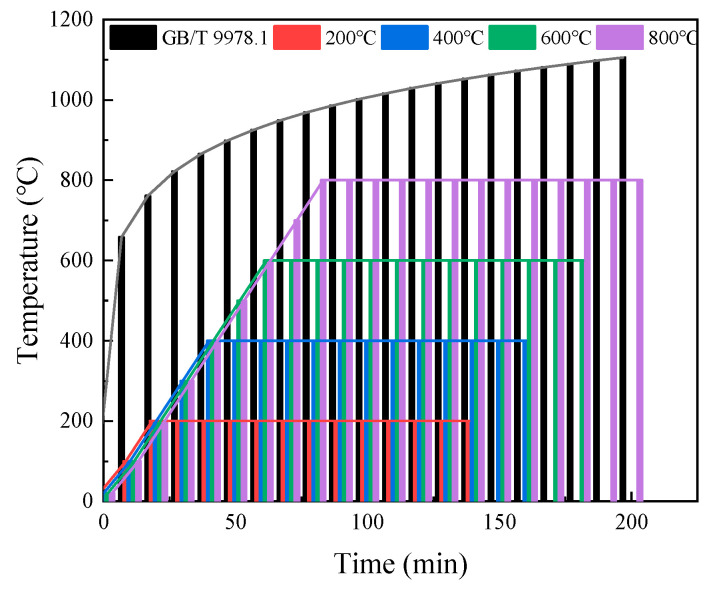
Standard heating curve and actual heating curve.

**Figure 2:**
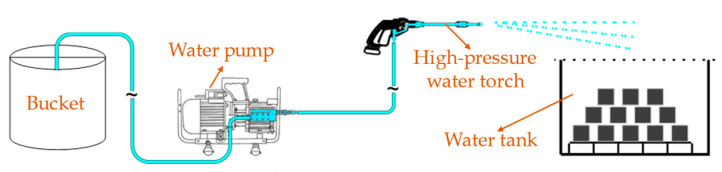
Schematic diagram of water spray device.

A high temperature trolley furnace was selected as the heating equipment. In accordance with the experimental requirements, the specimens were placed into a high temperature trolley furnace at a room temperature of 20 °C. Then, the heating speed was set to 10 °C per minute, and the target temperatures were 200 °C, 400 °C, 600 °C, and 800 °C, respectively. The temperature of the furnace began to rise immediately through the gradual heating of the resistance wire, and upon reaching the predetermined temperature, a constant temperature treatment was conducted for a duration of 120 min to ensure that the temperature on the surface and inside of the specimen was consistent with the environment’s temperature. After the constant temperature treatment was finished, the furnace was turned off to complete the entire heating process. The standard and actual temperature rise curves are given in Figure 1. The specimens subjected to high temperatures are categorized into two groups (Group A and Group B). The specimens in Group A underwent natural cooling. Once the furnace reached the desired temperature and completed the constant temperature treatment, the furnace door was opened, allowing for the sample to cool down to room temperature naturally. Subsequently, the sample was removed from the furnace. On the other hand, the specimens in Group B used water spray cooling. The specimens were removed from the furnace with a clamp once the test was completed and placed in a container for water spray treatment. The schematic diagram of the water spray device is depicted in Figure 2.

The amount of water spray was determined in accordance with the provisions of outdoor fire hydrant fire water spray in Chinese standard GB 50974-2014 [27], as well as the principles of the water volume in the unit area. Therefore, the water spray time in this study can be calculated as follows:

(1) $$\frac{Q_{2}T_{2}}{A}=\frac{\eta Q_{1}T_{1}}{\pi R^{2}}$$

in which *Q*_1_ and *Q*_2_ are water consumption of fire extinguishing and testing, respectively (L/s). *T*_1_ and *T*_2_ are water spray time of fire extinguishing and testing, respectively (h). *A* is the cross-sectional area of bucket (m^2^), *R* is coverage radius of fire sprinkler (m), and *η* is the reduction coefficient of water belt bending and head loss.

### 2.3. Mechanical Property Test

#### 2.3.1. Compressive Strength Remaining in Specimens following Exposure to High Temperatures

In this experiment, a 2000 kN microcomputer-controlled electro hydraulic servo universal testing machine from the Shaanxi Key Laboratory of Concrete Structure Safety and Durability was used for loading tests. A total of 25 groups were designed, with 3 specimens in each group and a total of 75 specimens. Based on the relevant provisions of Chinese standards GB/T 50081 2019 [28], the test adopted uniform and continuous loading with a loading rate of 0.8 MPa/s, and the size conversion coefficient was 0.9. Therefore, the compressive strength is calculated as follows:

(2) $$f_{cc}=0.9\times \frac{F}{A}$$

in which *f*_cc_ is the compressive strength of concrete specimen (MPa), *F* is the failure load of specimen (N), and *A* is specimen bearing area (mm^2^).

#### 2.3.2. Residual Flexural Strength of Specimens after High-Temperature Action

The testing of flexural strength was also conducted in accordance with the requirements of [28]. A prism specimen with a side length of 100 mm × 100 mm × 400 mm was used to design 25 groups of specimens (three specimens in each group); thus, a total of 75 specimens could be used for the testing of flexural strength. The tests were carried out with continuous uniform loading at a rate of 0.08 MPa/s. If the fracture surface of the specimen is outside the two loading lines of concentrated load, the test results are void. When the fracture point of the lower edge of the specimen is located between the two loading lines of concentrated load, the calculation of the specimen’s flexural strength can be determined using Equation (3):

(3) $$f_{t}=\frac{Fl}{bh^{2}}$$

in which *f*_t_ is flexural strength of fiber-reinforced concrete (MPa), *F* is failure load of specimen (N), *l* is span between supports (mm), and *b* and *h* are specimen section width and height of the specimen (mm), respectively.

## 3. Test Results and Analysis

### 3.1. Appearance Form

**Figure 3:**
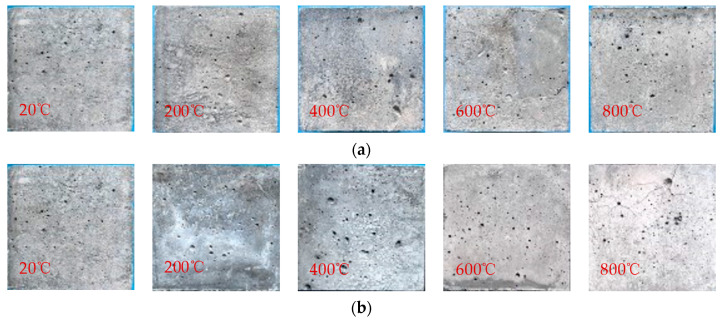
Apparent diagram of specimens after high temperature: (**a**) natural cooling; (**b**) water spray cooling.

Considering that the fiber content has little influence on the appearance of the specimen, the observation focused on the appearance characteristics of HSC specimens with 0.2% PVA fiber content, which underwent continuous changes as the heating temperature increased. The apparent morphological changes in PVA fiber-reinforced HSC using different cooling methods after high-temperature action are shown in Figure 3. As the temperature rises, the specimen’s color shifts from dark greenish grey to off-white. At room temperature, the color is greenish grey. At a temperature of 200 °C, the naturally cooling specimen’s color stays the same, but the water-sprayed one is dark greenish grey. Upon reaching a temperature of 400 °C, the natural cooling specimen turns light yellow, while the water spray cooling one remains unchanged. Upon heating to 600 °C, both specimens become grey, and the water spray cooling one being a lighter shade. Finally, when the temperature reaches 800 °C, the color is white after water spray cooling. Additionally, numerous staggered cracks become visible on the surface, and significant peeling occurs, while the color of the naturally cooling specimen remains similar to that observed at 600 °C.

At room temperature, the specimen contains free water, and its surface is greenish grey. When the temperature increases to 200 °C, the free water and a small amount of bound water in the specimen are lost, while the amount of gel decomposition is not large, so its surface color does not change significantly. When the temperature reaches 400 °C, the bound water inside the specimen further evaporates, and the C-S-H crystal begins to change. The color of the specimen using natural cooling changes from bluish grey to light yellow, while the color changes of specimen using water spray cooling is not significant due to the reaction between the internal impurities and water. When the temperature is 600 °C, Ca(OH)_2_ begins to dehydrate and decompose. After natural cooling action, the main components are CaO, SiO_2_, etc., so the color appears grayish white. However, after the action of water spray cooling, the color change rate in the test block is faster than that at 400 °C due to the water rust reaction. When the heating temperature is 800 °C, the gel inside the concrete is almost completely changed, and the color is white after cooling by spraying water. It can be seen that the changes in the apparent phenomena of fiber-reinforced concrete after high temperature are directly related to the changes in its internal components.

### 3.2. Mass Loss Rate

#### 3.2.1. The Mass Loss Rate of Specimens Using Different Cooling Methods

After removing the specimen from the furnace or cooling device, it was first dried in a drying oven for 24 h and weighed; then, the specimens were weighed again 24 h later, and the final mass of the specimen after cooling was determined once there was no change in mass.

Based on the mass changes observed in the specimens before and after exposure to high temperatures, the mass loss rates values are detailed in Table 3, which also includes the standard deviation (SD) and coefficient of variation (CV). Although certain groups exhibit slightly higher CV values, given the intricate nature of high-temperature effects and cooling methods on concrete performance, the overall results are deemed reliable. The same phenomenon can also be found in the test results of dynamic elastic modulus and mechanical strength.

#### 3.2.2. Influence of Heating Temperature on Mass Loss Rate

**Figure 4:**
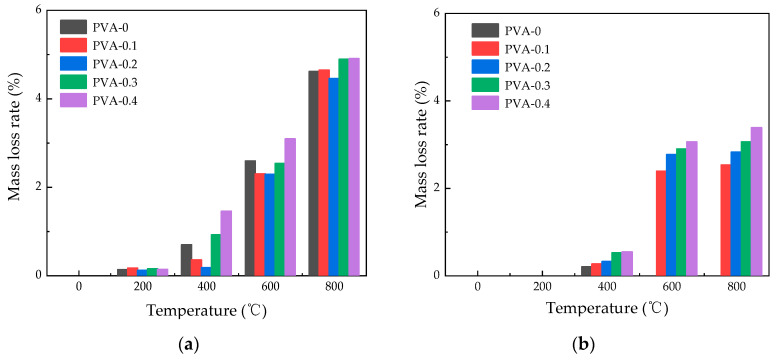
Change in mass loss rate of specimens with different heating temperatures: (**a**) change in mass loss rate with temperature under natural cooling; (**b**) change in mass loss rate with temperature under water spray cooling.

Figure 4 illustrates how the rate of mass loss for PVA fiber-reinforced HSC varies with temperature following exposure to elevated temperatures. It is evident from the figure that the rate of mass loss escalates as the temperature climbs. As temperature rises from 20 °C to 400 °C, the fiber-reinforced concrete’s mass loss rate climbs steadily. However, above 400 °C, the rate of mass loss rises steeply in response to further increases in temperature. Under natural cooling conditions, the mass loss rates at 600 °C are 2.692%, 2.808%, 2.94%, and 3.59% for 0.1%, 0.2%, 0.3%, and 0.4% content of PVA fibers, respectively. Under water spray cooling, the corresponding mass loss rates are 2.39%, 2.77%, 2.90%, and 3.06%, respectively. At 800 °C, the peak mass loss rate for natural cooling reaches 5.25%, while for spray cooling ones, it is lower at 3.38%. This phenomenon occurs because when the temperature surpasses 400 °C, the fibers undergo complete melting, leading to the formation of irregular pores and cracks. As a result, the mass loss rate of specimens escalates with the ongoing propagation and expansion of cracks [29]. It should be pointed out that the specimens cooled by water spray did not show significant mass changes at 200 °C, which may be due to the fact that the moisture absorbed by the specimens was exactly equal to the mass loss. It could be observed that there is a linear increase in mass loss with increasing content in Figure 4b, while Figure 4a does not show the same trend. This may be due to the fact that under the action of water spray cooling, the contact area between the concrete and water increases with the increase in fiber content, resulting in a gradual increase in mass loss rate.

#### 3.2.3. Effect of Fiber Content on Mass Loss Rate

**Figure 5:**
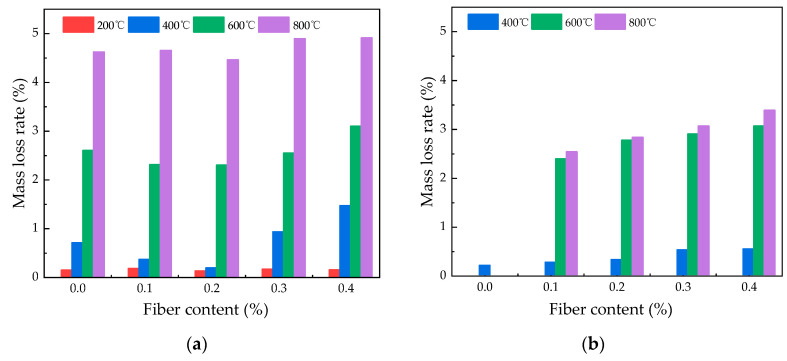
Change in mass loss rate of specimens with different fiber contents: (**a**) change in mass loss rate with fiber content under natural cooling; (**b**) change in mass loss rate with fiber content under water spray cooling.

Figure 5 shows the mass loss rates of high-temperature-exposed specimens with different PVA fiber amounts. Under water spray cooling, higher fiber content leads to increased mass loss rates in HSC, which is still less than that under natural cooling. For temperatures between 20 °C and 200 °C, the mass loss rates for all fiber contents under both cooling methods are below 0.2%, indicating a limited effect of fiber content on mass loss at lower temperatures. Under natural cooling conditions, between temperatures of 400 °C and 800 °C, the sample with a fiber content of 0.2% exhibits the lowest mass loss, with values of 0.18%, 2.29%, and 4.46%, respectively. Under water spray cooling, at a temperature of 400 °C, the mass loss rates are 0.27%, 0.32%, 0.52%, and 0.54% for fiber contents of 0.1%, 0.2%, 0.3%, and 0.4%, respectively. When the specimens are heated to 800 °C, specimens with 0.2% and 0.3% fiber content show mass loss rates of 2.83% and 3.06%, respectively. 

The results indicate that the mass loss rate of specimens with fiber content of 0.2% is lower than other specimens under the action of natural cooling, while for the specimens using water spray cooling method, the mass loss rate under the action of different temperatures increases slowly as the fiber content increases. Therefore, it can be concluded that the optimum fiber content for the mass loss rate is 0.2%.

### 3.3. Dynamic Elastic Modulus

This paper used and ultrasonic method to test the damage of specimens after exposure to high temperature following different cooling methods. The dynamic elastic modulus values are detailed in Table 4, which also includes the SD and CV.

Currently, the ultrasonic method is widely employed as a nondestructive testing technique for concrete. By measuring the P-wave and S-wave velocities, the dynamic elastic modulus of the concrete can be ascertained [30]. Measuring the ultrasonic velocity of concrete provides a better understanding of its internal structure. Ultrasonic velocity measurements are used to determine dynamic elastic modulus of the concrete, which assess damage post-high-temperature exposure [31]. An increase in the dynamic elastic modulus leads to a denser concrete interior, which in turn enhances its strength. During the test process, two opposite non-poured surfaces were used as test surfaces, diagonal lines were drawn on them, and the intersection points of the diagonal lines were used as ultrasonic detection points. To minimize the friction effect at the interface and reduce testing errors, it is essential to apply a layer of petroleum jelly on the surface of both non-metallic ultrasonic probes and specimens [20].

**Figure 6:**
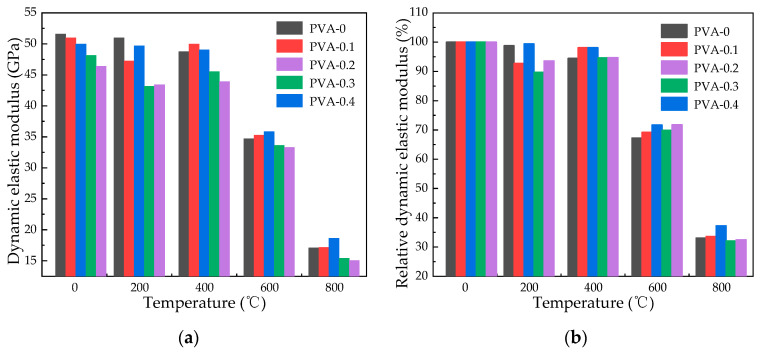
Dynamic elastic modulus of specimens after natural cooling: (**a**) dynamic elastic modulus of specimens after natural cooling; (**b**) relative residual dynamic elastic modulus of specimens after natural cooling.

**Figure 7:**
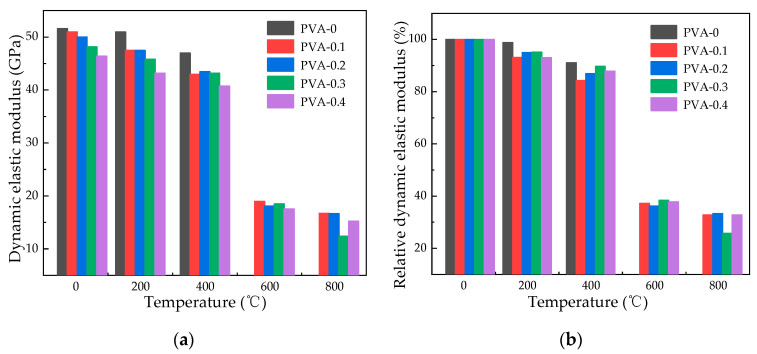
Dynamic elastic modulus of specimens after water spray cooling: (**a**) dynamic modulus of elasticity of specimens after water spray cooling; (**b**) relative residual dynamic elastic modulus of specimens after water spray cooling.

Figure 6 and Figure 7 present the dynamic elastic modulus of specimens subjected to high heat and then cooled naturally or by water spray, showing how the dynamic elastic modulus and relative residual dynamic elastic modulus change as temperature increases. At a temperature of 200 °C, the relative dynamic elastic modulus of specimens subjected to natural cooling with fiber contents of 0%, 0.1%, 0.2%, 0.3%, and 0.4% decreases by 1.24%, 7.27%, 0.63%, 10.33%, and 6.39%, respectively, while that of water spray cooling decreases by 1.24%, 6.87%, 5.06%, 4.82%, and 6.94%, respectively. At a temperature of 400 °C, the relative dynamic elastic modulus of specimens subjected to natural cooling decreases by 5.54%, while that of specimens subjected to water spray cooling decreases by 15.68%. At a temperature of 600 °C, the relative dynamic elastic modulus decreases by a maximum of 32.73% for specimens subjected to natural cooling, while for specimens subjected to water spray cooling, the decrease is as high as 63.83%. Pan [32] concluded that a rise in void ratio results in a reduction in ultrasonic speed. Hence, when fiber-reinforced concrete is water spray cooled following exposure to high temperatures, there is a significant decrease in the relative dynamic elastic modulus. At a heating temperature of 800 °C with a fiber content of 0.3%, the specimen’s relative residual dynamic elastic modulus stands at 32.06% following natural cooling, while after water spray cooling, it drops to 25.72%.

### 3.4. Compressive Strength

#### 3.4.1. The Compressive Strength Specimens Using Different Cooling Methods

The compressive strength values are given in Table 5, which also include the SD and CV.

#### 3.4.2. Effect of Temperature on Compressive Strength

**Figure 8:**
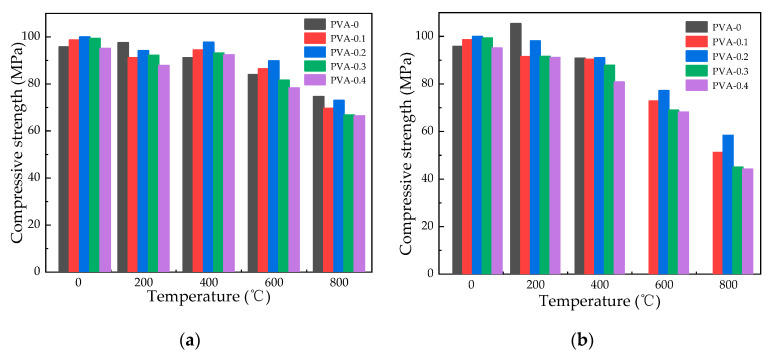
Changes in compressive strength with different temperatures: (**a**) changes in compressive strength at various temperatures under natural cooling; (**b**) changes in compressive strength with different temperatures under water spray cooling.

The data presented in Figure 8 indicate a decline in the concrete’s residual compressive strength with rising temperatures. A residual strength increase of 10% is noted in specimens with water spray cooling at 200 °C, in contrast to a slight 1.8% enhancement with natural cooling. When the temperature is higher than 400 °C, there is a noticeable reduction in compressive strength. This phenomenon occurs because when hydrated cement clinker is at 600 °C, hydration products decompose, and compressive strength drops sharply [33]. When the temperature is 400 °C, the compressive strength of 0.2% fiber-reinforced concrete under different cooling is 97.74 MPa and 91.00 MPa, respectively, which slowly decreases compared with the compressive strength at 200 °C. When the temperature is higher than 400 °C, the compressive strength changes from 92.79 MPa to 72.99 MPa when the temperature is 600 °C and 800 °C under natural cooling, while it changes from 77.18 MPa to 58.32 MPa under water spray cooling, suggesting a noticeable impact of temperature on compressive strength. It is observed that the compressive strength achieved through natural cooling surpasses that attained through water spray cooling. This finding aligns with the outcomes reported by Jing et al. [18] and Li [19].

#### 3.4.3. Effect of Fiber Content on Compressive Strength

**Figure 9:**
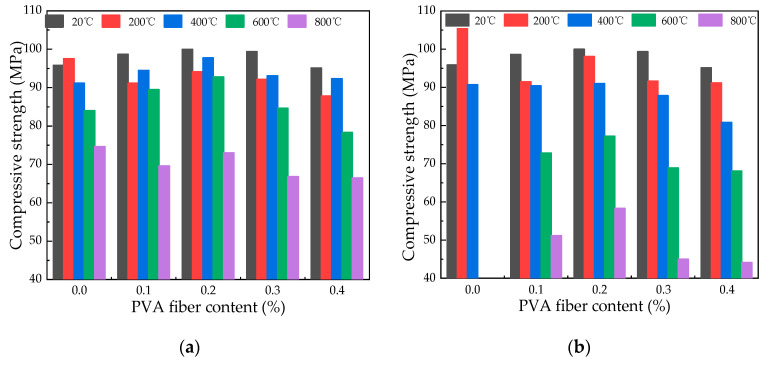
Compressive strength fluctuation with fiber content: (**a**) compressive strength changes with fiber content during natural cooling; (**b**) compressive strength changes with fiber content under water spray cooling.

Figure 9 displays a trend in specimens containing escalating amounts of PVA fiber, where an initial increase in compressive strength is succeeded by a subsequent decrease. At ambient temperature, the incorporation of 0.1% to 0.2% fiber content led to an augmentation in compressive strength of about 3% to 6% relative to plain HSC. Conversely, elevating the fiber content to 0.3% and 0.4% resulted in a reduction in compressive strength by an estimated 10%. The reason for this change is because the softening and stiffness of fibers are caused by high-temperature exposure, reducing strength when added [34]. When the fiber content is 0.1%, 0.2%, 0.3%, and 0.4%, the average compressive strength of natural cooling at different temperatures is 88.67 MPa, 91.53 MPa, 87.20 MPa, and 84.00 MPa, respectively. The average compressive strength of water spray cooling at different temperatures is 79.67 MPa, 84.92 MPa, 78.55 MPa, and 74.14 MPa, respectively. Therefore, compressive strength peaks at 0.2% PVA fiber content.

#### 3.4.4. Relation between Compressive Strength and Heating Temperature

**Figure 10:**
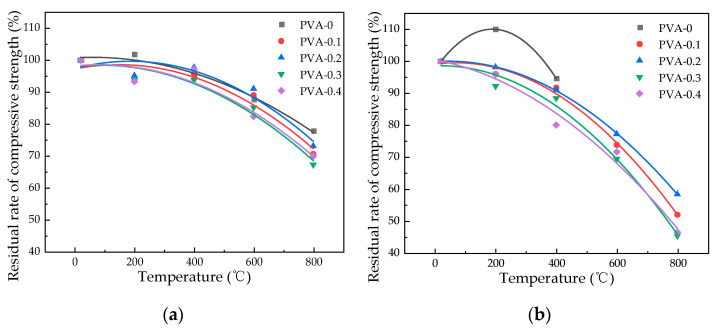
Fitting curve graph of compressive strength: (**a**) fitted curve of compressive strength under natural cooling; (**b**) fitted curves of compressive strength under water spray cooling conditions.

A correlation was formulated to apply the law describing how specimen compressive strength varies with temperature to engineering practices. This correlation connects the remaining compressive strength of specimens after high-temperature exposure, as represented by the fitting curve in Figure 10.

Equation (4) serves as an approximation tool for predicting the variation in residual compressive strength of specimens, taking into account differing fiber content and temperatures:

(4) $$\frac{f_{cu}^{T}}{f_{cu}}=A+BT+CT^{2}$$

in which $f_{cu}^{T}$ is the compressive strength of the specimens after high-temperature action (MPa), $f_{cu}$ is the compressive strength of specimens at room temperature (MPa), *T* is the design temperature (20 °C~800 °C), and A, B, and C are unknown parameters.

The specific values are shown in Table 6.

### 3.5. Flexural Strength

#### 3.5.1. Flexural Strength of Specimens Using Different Cooling Methods

The flexural strength values are shown in Table 7, which also include SD and CV.

#### 3.5.2. Influence of Heating Temperature on Flexural Strength

**Figure 11:**
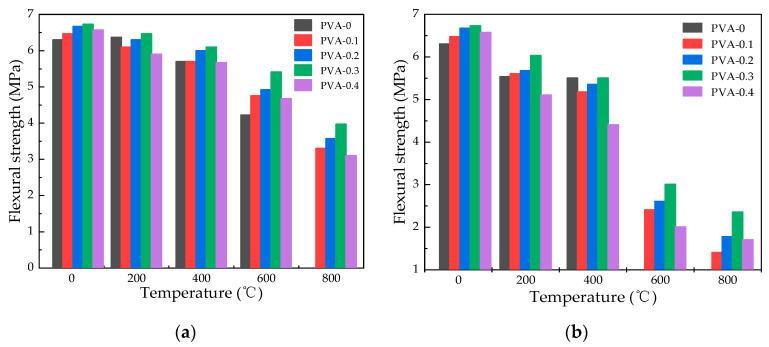
Flexural strength variation in specimens at different temperatures: (**a**) flexural strength changes under natural cooling; (**b**) flexural strength changes under water spray cooling.

Figure 11 illustrates a decrease in flexural strength for specimens with varying fiber contents as the heating temperature increases. Under natural cooling conditions, when the heating temperature is 200 °C, 400 °C, 600 °C, and 800 °C, the flexural strength of specimen with fiber content of 0.3% concrete is 6.47 Mpa, 6.10 MPa, 5.41 MPa, and 3.97 Mpa, respectively. The decrease in flexural strength was increased by 3.86%, 5.50%, 10.22%, and 21.43%, respectively, with respect to plain HSC. Water spray cooling led to a decrease in flexural strength of concrete with 0.2% fiber content from 10.40% to 7.88% and from 5.67 MPa to 5.35 MPa as temperature rose from 200 °C to 400 °C. Furthermore, the reduction in flexural strength accelerated once the temperature surpassed 400 °C, decreasing from 37.14% to 9.66%. The average value of the remaining flexural strength after natural cooling is 3.49 MPa and after water spray cooling is 1.81 MPa at the temperature of 800 °C. The largest reduction in flexural strength is observed at a temperature of 800 °C, and natural cooling has a more pronounced effect compared to water spray cooling.

#### 3.5.3. Effect of Fiber Content on Flexural Strength

**Figure 12:**
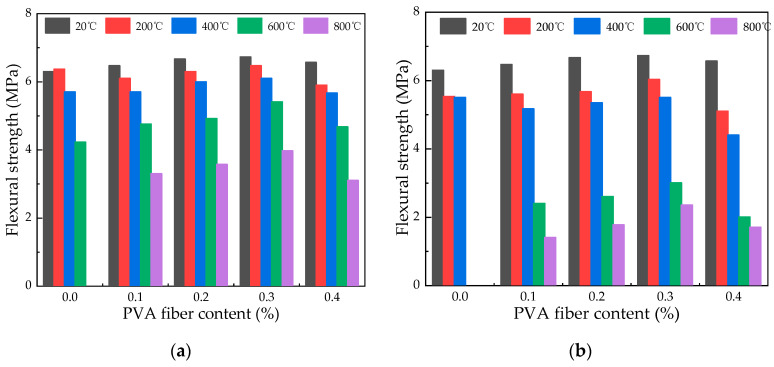
Flexural strength variation with different fiber contents in specimens: (**a**) effect of varying fiber contents on the flexural strength in specimens under natural cooling; (**b**) impact of fiber content variation on flexural strength in water spray cooled specimens.

As shown in Figure 12, it is possible to notice a distinct pattern where the flexural strength experiences an initial increment followed by a decrement as the fiber content is heightened. At room temperature, the inclusion of PVA fibers in quantities of 0.1%, 0.2%, 0.3%, and 0.4% results in an increase of 2.71%, 5.95%, 6.88%, and 4.34%, respectively. When the temperature changes from 400 °C to 600 °C, the flexural strength of 0.3% fiber content under natural cooling conditions changes from 5.41 MPa to 3.97 MPa, while the values of water spray cooling changes from 3.00 MPa to 2.35 MPa. A researcher found that when the fiber content increases, the pores in the concrete structure increase, which causes a decrease in strength at high temperatures [35]. With 0.3% PVA fiber content, the average flexural strength under natural cooling is 5.74 MPa, compared to 4.72 MPa for water spray cooling, indicating that PVA fibers enhance the specimens’ resistance to flexural stress. Considering the flexural strength of specimens under natural and water spray cooling conditions, the optimal fiber content for compressive strength is determined as 0.3%.

#### 3.5.4. Relation between Flexural Strength and Heating Temperature

**Figure 13:**
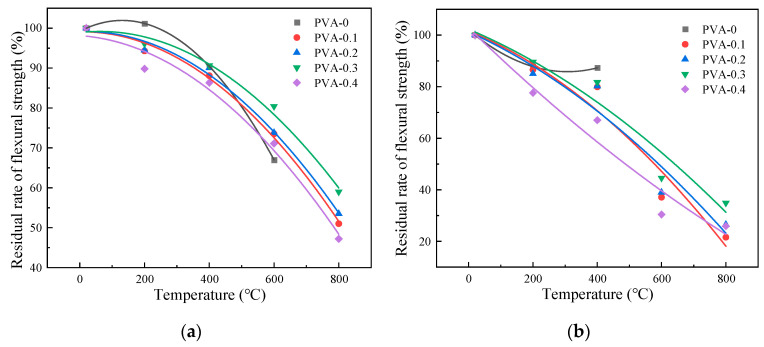
Fitting curve graph of flexural strength: (**a**) fitting curve of natural cooling; (**b**) fitting curve of water spray cooling.

This research examines how various cooling methods influence the residual rate of flexural strength, which is the ratio of high-temperature flexural strength to that at normal temperatures, as determined by analyzing the curve in Figure 13 for specimens exposed to high temperatures.

Under different cooling methods, the specific expression of the residual rate of flexural strength of specimens with different fiber content changes with temperature is shown in Equation (5):

(5) $$\frac{f_{f}^{T}}{f_{f}}=A+BT+CT^{2}$$

in which $f_{f}^{T}$ is the flexural strength of specimen after high temperature action (MPa), $f_{f}$ is flexural strength of specimen at room temperature (MPa), *T* is the design temperature (20 °C~800 °C), and A, B, and C are unknown parameters in the formula.

The specific values are shown in Table 8.

### 3.6. Scanning Electron Microscope

PVA fiber melts due to high temperature [36]; thus, when the temperature is lower than the melting point of the fiber, the fiber state will not change. At 300 °C, the fiber undergoes melting, and hydrated calcium silicate (C-S-H) interlayer water is released. At the same time, part of the chemical binding water of hydrated sulfoaluminate in the concrete is lost, and the cement slurry further loses water due to Ca(OH)_2_ decomposition. The material inside the concrete specimen gradually becomes loose from the original dense structure, which reduces the strength. At the same time, the high temperature causes fiber ablation and generates capillary channels, which provide space for the release of steam pressure; this diminishes the steam pressure resulting from the movement of water and gases and lessens the harm inflicted by steam tension within the concrete [37]. The primary cause of thermal degradation in cement slurry at elevated temperatures is the interfacial microcracking that occurs between dehydrated cement particles and the slurry’s matrix, accompanied by alterations in the microstructure of the C-S-H [38]. When the concrete is exposed to high temperatures, voids and fissures may develop within it.

#### 3.6.1. Changes in the Microstructure of HSC at Various Temperatures

**Figure 14:**
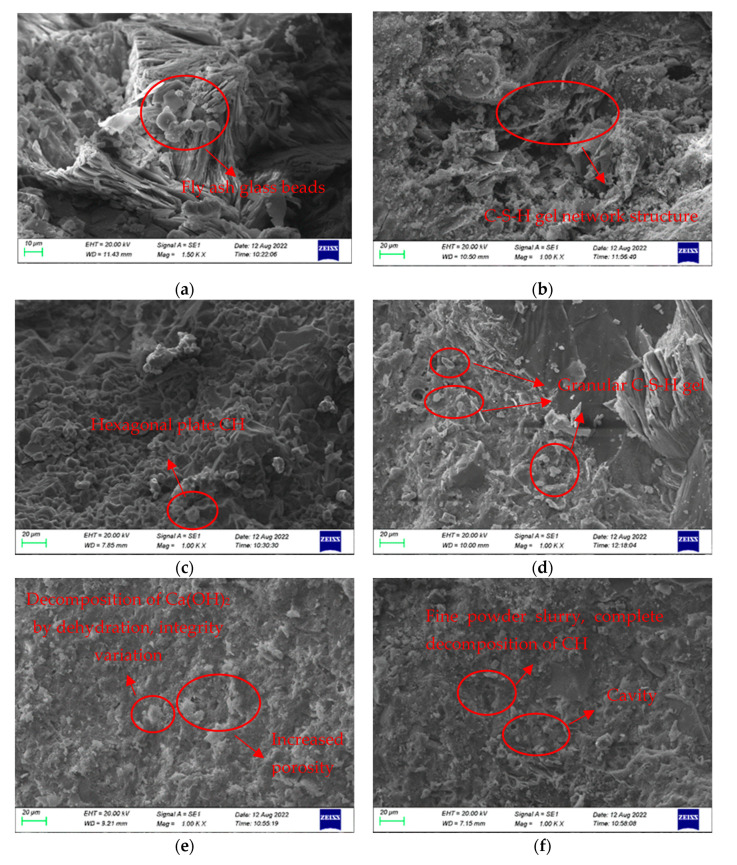
Cement paste at different temperatures: (**a**) unhydrated fly ash glass beads at 20 °C; (**b**) C-S-H gel network structure at 20 °C; (**c**) C-S-H gel is decomposed by heat at 200 °C; (**d**) granular C-S-H gel at 400 °C; (**e**) dehydration decomposition of Ca(OH)_2_ at 600 °C; (**f**) complete decomposition of Ca(OH)_2_ at 800 °C.

Electron microscope scanning photos of HSC cement slurry after different heating temperatures are shown in Figure 14. At normal temperature, the cement slurry in the concrete has a complete structure and tight bonding, the fly ash glass beads are not hydrated, and the hexagon plate calcium hydroxide (CH) is difficult to distinguish due to being deposited in C-S-H gel, as shown in Figure 14a,b. When the heating temperature is 200 °C, the C-S-H gel is decomposed by the heat, and the overall structure is basically intact, but it begins to show signs of loosening, as seen in Figure 14c. At a heating temperature of 400 °C, the structure of cement hydration paste exhibits an obvious loosening phenomenon, the C-S-H changes from dense radial to looser flocculent small particles, the hydration is greatly reduced, and the cracks are obviously increased, which can be seen in Figure 14d. When the heating temperature is 600 °C, extensive dehydration and decomposition of Ca(OH)_2_ occur, which dismantle the C-S-H gel network structure and significantly enlarging the volume of the pores, thus substantially reducing compressive strength, as given in Figure 14e. By the time the heating temperature is 800 °C, Ca(OH)_2_ has completely decomposed [39], numerous fractures and holes appear, and the overall shape is a loose honeycomb. The breakdown of Ca(OH)_2_ and CaCO_3_ produces gas, resulting in numerous tiny perforations in the cement mixture and causing the mixture to become powdery. The fractures on the surface of the sample have penetrated fully, the width gradually increases, and finally, the external layer of skin crumbles away, as shown in Figure 14f. A significant decrease of 54.7% in compressive strength was observed following the completion of water spray cooling.

#### 3.6.2. Variation in PVA Concrete Fibers and Melting Pores with Different Temperatures

In this paper, microstructural changes in 0.2% PVA fiber reinforced HSC were studied at different heating temperatures (20 °C, 200 °C, 400 °C, 600 °C, and 800 °C), and the microstructural changes in cement slurry and fiber melting channels were analyzed by SEM.

**Figure 15:**
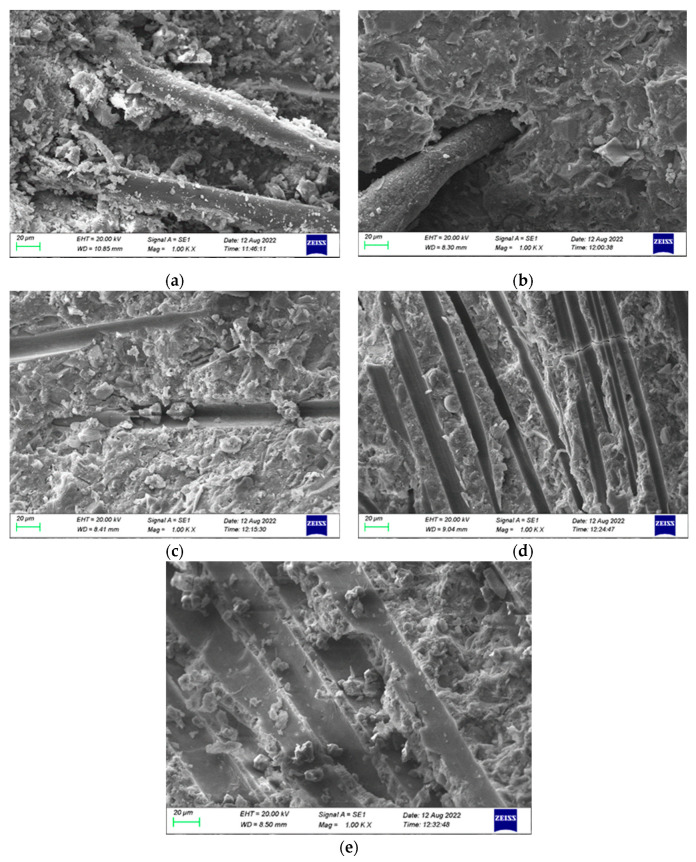
Fibers and fiber melting channels at different temperatures: (**a**) PVA fiber tightly encapsulated in cement paste at 20 °C; (**b**) the volume of concrete begins to expand at 200 °C; (**c**) PVA fiber melted at 400 °C; (**d**) staggered distribution of channels at 600 °C; (**e**) cracks increase at 800 °C, microstructure similar to that of plain HSC.

Based on the SEM analysis of the PVA fiber in HSC subjected to elevated temperatures, as shown in Figure 15, the following outcomes could be concluded: at room temperature, a significant accumulation of hydration products could be observed on the surface of the PVA fiber, which were tightly connected with the cement paste, which wrapped the PVA fiber tightly; the bond between fiber and concrete in structures can be improved to ensure greater durability and strength. As temperatures rose, the volume expansion of the concrete was mitigated by the bridging effect of fibers with the concrete, up until the melting point of the fiber. After that, the fiber created tiny channels in the cement matrix [40], which facilitated the emission of atmospheric moisture, reduced the pressure of the vapor within the specimen, and prevented the concrete from bursting and developing cracks, contributing to its enhanced durability and resistance. Once the temperature exceeded 400 °C, the occurrence of cracking intensified, resulting in a microstructure of PVA fiber-reinforced HSC that was similar to that of plain HSC. Therefore, integrating PVA fibers may enhance the structural properties of concrete, yet there are some constraints at elevated temperatures.

From the above analysis, it can be observed that as the temperature increases, the composition of HSC changes, resulting in an increase and then a decrease in the mechanical strength, while for the fiber-reinforced HSC, at low temperatures, the melting of fibers creates pores in the concrete, which reduce the occurrence of microcracks in the cement paste. However, at high temperatures, cement paste without fibers also cracks, ultimately leading to a decrease in mechanical strength.

## 4. Conclusions

This study examined how different PVA fiber amounts and cooling methods affect the physical and mechanical features of HSC, such as appearance, mass loss, elastic modulus, and compressive and flexural strength. Additionally, SEM analysis was conducted to investigate the microstructure of fiber-reinforced concrete subjected to elevated temperatures. The primary conclusions are given as follows:

(1) With increasing temperature, the appearance of the specimen changed, shifting in color from dark cyan gray to gray white, with more severe surface damage and an increasing rate of mass loss. Specimens that underwent water spray cooling exhibited a higher degree of surface damage relative to those that cooled naturally.
(2) As the heating temperature rose, the dynamic elastic modulus of specimens with different PVA fiber amounts consistently fell, with a progressively sharper rate of decline. At a heating temperature of 800 °C, the specimens exhibited a residual dynamic elastic modulus of 32.06% after natural cooling, in contrast to 25.72% with water spray cooling.
(3) The residual strength dwindled with higher temperatures, with compressive strength falling as temperature increased. The peak compressive strength occurred at 0.2% PVA fiber content. Moreover, natural cooling preserved higher compressive strength compared to water spray cooling.
(4) Specimen flexural strength declined with increasing temperature. Fiber content first increased but then reduced in strength, peaking at 0.3% PVA. At a temperature of 800 °C, natural cooling resulted in a residual flexural strength of 3.97 MPa, versus 2.35 MPa with water spray cooling.
(5) It was found by scanning electron microscope that at higher temperatures, the pores in the concrete expanded, cracks continued to develop, and the connection between aggregate and cement mortar gradually separated, forming a large space. As a consequence, the concrete’s ability to withstand compression steadily diminished.

## Figures and Tables

**Table 1:** Mix ratio of HSC.

Water (kg/m^3^)	Cement (kg/m^3^)	Fly Ash (kg/m^3^)	Slag (kg/m^3^)	Silica Fume (kg/m^3^)	Sand (kg/m^3^)	Coarse Aggregate (kg/m^3^)	Water Reducer (kg/m^3^)	Volume Content of PVA Fiber (%)
123	461.25	61.5	61.5	30.75	650.56	1070.44	4.92	0
123	461.25	61.5	61.5	30.75	650.56	1070.44	4.92	0.1
123	461.25	61.5	61.5	30.75	650.56	1070.44	4.92	0.2
123	461.25	61.5	61.5	30.75	650.56	1070.44	4.92	0.3
123	461.25	61.5	61.5	30.75	650.56	1070.44	4.92	0.4

**Table 2:** Groups for testing the performance of specimens.

Temperature	PVA Fiber Content	Cooling Type	Physical Performance	Mechanical Property Index	Number of Each Group
20 °C	0~0.4% (Level 5, amplitude 0.1%)	Natural cooling and water spray cooling	Apparent change, mass loss rate, and micro-analysis	Dynamic elastic modulus, compressive strength, and flexural strength	Three
200 °C					
400 °C					
600 °C					
800 °C					

**Table 3:** Mass loss rate of specimens using different cooling methods after high-temperature action.

Cooling Method	Fiber Content (%)	Mass Loss Rate: AV (MPa), CV (%)														
		20 °C			200 °C			400 °C			600 °C			800 °C		
		AV	SD	CV	AV	SD	CV	AV	SD	CV	AV	SD	CV	AV	SD	CV
Natural cooling	0	0	-	-	0	-	-	0.132	0.02	0.16	2.463	0.42	0.17	4.853	0.63	0.13
	0.1	0	-	-	0.133	0.04	0.28	0.200	0.03	0.14	2.692	0.32	0.12	4.899	0.69	0.14
	0.2	0	-	-	0.069	0.01	0.15	0.335	0.05	0.15	2.808	0.45	0.16	4.637	0.51	0.11
	0.3	0	-	-	0.066	0.01	0.15	0.332	0.04	0.11	2.935	0.41	0.14	5.014	0.75	0.15
	0.4	0	-	-	0.067	0.01	0.16	0.336	0.04	0.11	3.589	0.39	0.11	5.254	0.89	0.17
Water spray cooling	0	0	-	-	0	-	-	0.202	0.04	0.18	-	-	-			
	0.1	0	-	-	0	-	-	0.266	0.05	0.19	2.389	0.50	0.21	2.530	0.53	0.21
	0.2	0	-	-	0	-	-	0.321	0.05	0.17	2.770	0.53	0.19	2.526	0.45	0.18
	0.3	0	-	-	0	-	-	0.521	0.10	0.20	2.897	0.49	0.17	3.060	0.61	0.20
	0.4	0	-	-	0	-	-	0.540	0.10	0.18	3.060	0.58	0.19	3.382	0.64	0.19

**Table 4:** Dynamic elastic moduli of specimens using different cooling methods after high-temperature action.

Cooling Method	Fiber Content (%)	Dynamic Elastic Modulus: AV (MPa), CV (%)														
		20 °C			200 °C			400 °C			600 °C			800 °C		
		AV	SD	CV	AV	SD	CV	AV	SD	CV	AV	SD	CV	AV	SD	CV
Natural cooling	0	51.61	7.23	0.14	50.97	7.65	0.15	48.75	7.31	0.15	34.72	4.86	0.14	17.08	2.73	0.16
	0.1	50.97	5.61	0.11	47.27	6.62	0.14	50.00	8.00	0.16	35.28	4.94	0.14	17.14	2.57	0.15
	0.2	50.00	6.00	0.12	46.69	9.44	0.19	49.06	6.87	0.14	35.85	4.66	0.13	18.63	2.24	0.12
	0.3	48.15	7.22	0.15	43.18	6.48	0.15	45.55	6.38	0.14	33.64	5.38	0.16	15.44	2.32	0.15
	0.4	46.39	6.96	0.15	43.43	6.95	0.16	43.94	4.83	0.11	33.30	5.00	0.15	15.06	2.56	0.17
Water spraying cooling	0	51.61	7.74	0.15	50.97	6.63	0.13	46.98	7.52	0.16	-	-	-	-	-	-
	0.1	50.97	6.63	0.13	47.47	7.60	0.16	42.98	7.31	0.17	18.96	3.03	0.16	16.7	3.01	0.18
	0.2	50.00	6.50	0.13	47.47	8.07	0.17	43.45	7.82	0.18	18.09	3.08	0.17	16.64	3.16	0.19
	0.3	48.15	6.74	0.14	45.83	5.50	0.12	43.18	6.05	0.14	18.49	3.51	0.19	12.39	2.60	0.21
	0.4	46.39	5.57	0.12	43.18	6.91	0.16	40.75	6.93	0.17	17.54	3.51	0.20	15.21	2.43	0.16

**Table 5:** The compressive strength of specimens using different cooling methods after high-temperature action.

Cooling Method	Fiber Content (%)	Compressive Strength: AV (MPa), CV (%)														
		20 °C			200 °C			400 °C			600 °C			800 °C		
		AV	SD	CV	AV	SD	CV	AV	SD	CV	AV	SD	CV	AV	SD	CV
Natural cooling	0	6.30	0.57	0.09	6.37	0.76	0.12	5.70	0.74	0.13	4.22	0.59	0.14	-	-	-
	0.1	6.47	0.71	0.11	6.10	1.22	0.20	5.70	1.08	0.19	4.76	0.71	0.15	3.30	0.50	0.15
	0.2	0.67	1.00	0.15	6.30	1.01	0.16	6.00	1.02	0.17	4.92	0.54	0.11	3.57	0.50	0.14
	0.3	6.73	0.94	0.14	6.47	1.04	0.16	6.10	1.22	0.20	5.41	1.08	0.20	3.97	0.60	0.15
	0.4	6.57	1.12	0.17	5.90	0.77	0.13	5.67	0.85	0.15	4.67	1.07	0.23	3.10	0.37	0.12
Water spraying cooling	0	95.85	17.25	0.18	105.39	15.81	0.15	90.68	17.23	0.19	-	-	-	-	-	-
	0.1	98.64	14.80	0.15	85.32	19.62	0.23	90.41	17.18	0.19	72.81	13.11	0.18	51.17	9.21	0.18
	0.2	99.99	11.00	0.11	98.10	18.64	0.19	91.00	19.11	0.21	77.18	15.44	0.20	58.32	7.58	0.13
	0.3	99.36	21.86	0.22	91.62	15.58	0.17	87.84	19.32	0.22	68.94	9.65	0.14	45.00	9.45	0.21
	0.4	95.13	16.17	0.17	91.17	16.41	0.18	72.18	12.99	0.18	68.13	8.86	0.13	44.10	9.70	0.22

**Table 6:** Correlation parameters for temperature and residual compressive strength.

Cooling Type	PVA Fiber Content (%)	Fitting Parameters			Correlation Coefficient (Adjusted R^2^)
		A	B	C	
Natural cooling	0	100.7247	0.00501	−4.27698 × 10^−5^	0.97402
	0.1	97.2198	0.01824	−6.18422 ×10^−5^	0.82934
	0.2	97.3256	0.02481	−6.66056 × 10^−5^	0.84297
	0.3	97.9483	0.01021	−5.88231 × 10^−5^	0.91934
	0.4	98.2009	0.00905	−5.5638 × 10^−5^	0.86399
Water spray cooling	0	97.49357	0.1323	−3.4883 × 10^−4^	1.00000
	0.1	99.0554	0.01265	−8.94966 × 10^−5^	0.98919
	0.2	99.8766	−0.00639	−7.30479× 10^−5^	0.99978
	0.3	98.5374	0.00317	−8.64584× 10^−5^	0.97878
	0.4	100.4377	−0.01777	−6.03896× 10^−5^	0.96817

**Table 7:** The flexural strength of specimens using different cooling methods after high-temperature action.

Cooling Method	Fiber Content (%)	Flexural Strength: AV (MPa), CV (%)														
		20 °C			200 °C			400 °C			600 °C			800 °C		
		AV	SD	CV	AV	SD	CV	AV	SD	CV	AV	SD	CV	AV	SD	CV
Natural cooling	0	6.30	0.57	0.09	6.37	0.76	0.12	5.70	0.74	0.13	4.22	0.59	0.14	-	-	-
	0.1	6.47	0.71	0.11	6.10	1.22	0.20	5.70	1.08	0.19	4.76	0.71	0.15	3.30	0.50	0.15
	0.2	0.67	1.00	0.15	6.30	1.01	0.16	6.00	1.02	0.17	4.92	0.54	0.11	3.57	0.50	0.14
	0.3	6.73	0.94	0.14	6.47	1.04	0.16	6.10	1.22	0.20	5.41	1.08	0.20	3.97	0.60	0.15
	0.4	6.57	1.12	0.17	5.90	0.77	0.13	5.67	0.85	0.15	4.67	1.07	0.23	3.10	0.37	0.12
Water spraying cooling	0	6.30	0.95	0.15	1.38	0.25	5.50	5.50	0.99	0.18	-	-	-	-	-	-
	0.1	6.47	0.84	0.13	1.01	0.18	5.17	5.17	1.14	0.22	2.40	0.53	0.22	1.40	0.25	0.18
	0.2	6.67	1.07	0.16	0.62	0.11	5.35	5.35	0.80	0.15	2.60	0.47	0.18	1.77	0.37	0.21
	0.3	6.73	0.74	0.11	0.96	0.16	5.50	5.50	1.16	0.21	3.00	0.63	0.21	2.35	0.49	0.21
	0.4	6.57	1.25	0.19	0.77	0.15	4.40	4.40	0.75	0.17	2.00	0.32	0.16	1.70	0.32	0.19

**Table 8:** Parameters for residual flexural strength–temperature relationship fitting.

Cooling Type	PVA Fiber Content (%)	Fitting Parameters			Correlation Coefficient (Adjusted R^2^)
		A	B	C	
Natural cooling	0	99.209	0.04150	−1.58585 × 10^−4^	0.99999
	0.1	99.076	0.00114	−7.56218 × 10^−5^	0.99157
	0.2	89.112	0.00277	−7.44423 × 10^−5^	0.98812
	0.3	98.881	0.00904	−7.2109 × 10^−5^	0.98306
	0.4	98.126	−0.00561	−7.07223 × 10^−5^	0.96345
Water spray cooling	0	102.0427	−0.10557	1.71784 × 10^−4^	1.000000
	0.1	101.484	−0.04981	−6.79887 × 10^−5^	0.91146
	0.2	101.277	−0.05613	−5.18946 × 10^−5^	0.89419
	0.3	102.270	−0.05275	−4.49774 × 10^−5^	0.89637
	0.4	102.830	−0.12127	2.64135 × 10^−5^	0.91491

## Data Availability

The original contributions presented in the study are included in the article, further inquiries can be directed to the corresponding author.
